# A Manual of Practical Medical Electricity

**Published:** 1898-09

**Authors:** 


**A Manual of Practical Medical Electricity.** By DAWSON TURNER, M.D. Second Edition. Pp. xvi., 335. London: Baillière, Tindall and Cox. 1897.

**Practical Muscle Testing and the Treatment of Muscular Atrophies.** By W. S. HEDLEY, M.D. Pp. viii., 128. London: H. K. Lewis. 1897.

Any medical man who desires to gain a sufficient knowledge of electricity to enable him to use it intelligently in his daily practice will find Dr. Turner's book a very good one, and a student who carefully studies it will have a good elementary and practical working knowledge of electricity. It is clearly and scientifically written, and that it has attained the purpose for which it was intended is shewn by its having reached a second edition. The whole work has been revised, and a section added dealing with the Röntgen rays and their applications to medicine and surgery. Finally we ought to mention that the text is illustrated by a number of diagrams and good figures of apparatus, and also by a number of excellent skiagrams.

Dr. Hedley's book is also a good one and well-written. It has a smaller scope, as it pre-supposes an elementary knowledge of electricity and deals only with its practical medical applications in the testing of nerve and muscle reactions in the diagnosis of nervous diseases, and with the treatment of paralysis and muscular atrophies. The directions are all clearly given; the plan of the book is well thought out, and it seems likely to prove a useful one. There are some good photographs of the actions of various muscles, and the book will be serviceable for reference as to the action of a particular muscle. The directions for treatment are sound and practical, and the different modes of electrical treatment for different diseases are fully described: although it is pointed out that electricity when intelligently used in the manner best adapted for each case is capable of doing much good, more is not claimed for it as a means of cure than it is able to perform.

